# Evaluation of the infertile male – modern approach in the Procreation Medical Assistance (P.M.A.)

**Published:** 2012

**Authors:** Gratiela Draghici

**Affiliations:** Emergency University Hospital, Bucharest

**Keywords:** PMA, infertile couples, sperm, alimentation, ICSI

## Abstract

The infertility of the couple represents a constant preoccupation of the modern society; the supervision of the fertility of the population has become a necessity today, as a result of the behaviour and habitat modifications, which the modern life has generated.

Today the PMA (Assisted Human Reproduction) techniques allow the palliative solving of the medical problems, as far as the somatic plan is concerned, the incapacity of procreation of the couple.

However, beyond the attempt of practically solving infertility, at the world level, the specialists in the field of reproduction have projected evaluation and supervising couple fertility programs, trying to delineate the responsible factors of this reality.

## Introduction

The infertility of the couple represents a constant preoccupation of the modern society; the supervision of the fertility of the population has become a necessity today, as a result of the behaviour and habitat modifications, which the modern life has generated.

The fact that the spermatozoon has a role in the process of reproduction has been known for centuries, but its concrete contribution has been understood the moment Antoni Van Newenhoek has first described the “semen animalis” (spermatozoon) in ejaculation, in 1677.

Thus, the equal role of the spermatozoon and the oocyte has been accepted later in the process of reproduction, at the end of the 19th century. The understanding of the function and structure of the spermatozoon has been realized in the 20th century, as a result of the technological progress and the discovery of the DNA as a genetic material transmissible to the descendants.

Today the PMA (Assisted Human Reproduction) techniques allow the palliative solving of the medical problems, as far as the somatic plan is concerned, the incapacity of procreation of the couple.

However, beyond the attempt of practically solving infertility, at the world level, the specialists in the field of reproduction have projected evaluation and supervising couple fertility programs, trying to delineate the responsible factors of this reality.

In the study presented in the abstract, the infertile male has been rigorously evaluated with the purpose of identifying the following elements [**1**]:

1. potentially reversible causes;

2. ireversible causes which can be overcome with the help of assisted reproduction techniques by using the sperm of the partner;

3. ireversible conditions which cannot be solved by PMA and in which the couple is advised to appeal to the insemination with the sperm from the donor or even try to adopt;

4. significant medical pathology, the infertility being the manifestation of a severe pathology with a lethal potential (i.e. testicular tumors, hypophysis gland tumors);

5. genetic and/or chromosomal anomalies which can affect both the patient and the descendants, if the assisted reproduction techniques are used.

The investigation protocol of the infertile couple has presupposed the PRE-IVF balance, as a starting point. For the male, the pre-IVF balance has presupposed the following actions [**2**]:

I. Anamnesis – based on a QUESTIONNAIRE inspired from the “Research Program, Quality of sperm, Reproduction function, and Fertility in males”, initiated in the western Europe and structured in three major sections:

A. General questions – regarding the history of the life as a couple, the general behaviour of the person, the exposure to the environment factors which interfere with the reproduction function;

B. Questions regarding the genital and urinary antecedents;

C. Questions regarding the lifestyle, alimentation, and occupation.

II. The clinical examination in order to identify a possible severe pathology, which can be associated with male infertility.

III. The paraclinical examination:

1. The first evaluation of the quality of the sperm:

SPERMOGRAM Sperm culture Huhner Test

Sperm cytograme

repeated at three months

2. The second evaluation of the sperm quality:

AZOOSPERMIA TERATOSPERMIA ASTHENOSPERMIA

-bichemical markers - karyotype -biochemical markers

-hormone dosages -ultrastructure -antibodies identification

-karyotype -spermatozoon -functional tests

 -TESTICULAR BIOPSY-

3. Sperm functional tests [**3**]

a) Physical examination

 -Volume of the ejaculation=2-6 ml -hypospermia<2 ml

  -hyperspermia>2ml

 -Sperm liquefaction time=20-30 min.

 -Viscosity

 -ph=7,2-7,8

 -Odor

 -Whitish color

b) Count=20-200 mil/ml 0-AZOOSPERMIA

 <20 mil.-OLIGOSPERMIA

 >200 mil.-POLYSPERMY

c) Indicators of vitality and motility

 -vitality at 25 min.,2h,6h: >50%=normal

  <50%=NECROSPERMIA

 -mobility at 25 min.,2h,6h>40-50%=normal

  =0=AKINETOSPERMIA

  <40%=ASTENOSPERMIA

 =the character of mobility -progressive mobile

  -unprogressive mobile

d) Chemical resistance =%mobile sperm after 2h to ph 6,4

e) Abnormal forms >60%=TERATOSPERMIA

4. Biochemical markers

a) AZOOSPERMIA-secretory – Normal Carnitine

  -excretory – Low Carnitine

  FRU=0,Zn =N -occlusion of the ejaculatory canal

   -vesico-differential agenesis

  FRU=N,Zn=N-occlusion epididymis or canal

   Deferential

b) OLIGOAZOOSPERMIA-occlusion or unilateral agenesis FRU=low.

  -retrograde ejaculation FRU =low/0

  -prostatitis FRU=low/0

c) ASTENOSPERMIA –bilateral occlusion of the seminal vesicle of the cervical area

  FRU=0,Zn=N,CARNITINE=N

5. Hormonal dosages - FSH/LH,PROLACTINA.

**The purpose of the study:** the evaluation of the infertile male in a lot of 240 infertile couples.

**Study group:** The patients whose result in the sperm examination has proved the physical-chemical and biologically unsatisfactory parameters compared to the values accepted at an international level, thus selecting 22 couples who have benefited from ICSI from the 240.

### Results of the study

We have done studies regarding the following elements on the lot:

-distribution according to age groups of the ones who have presented for infertility;

-the cause that has determined the infertility: risk factors, antecedents of urinary infection, working in a toxic environment:

 =antecedents of testicular pathology;

 =the existence of some chronic diseases or long-term treatments;

 =the main etiologies met as the cause for secretory azoospermia;

-fecundability distribution;

-main I.C.S.I. indications

 After the data processing, we have obtained the following results:

1. **PLACE OF ORIGIN** -28,56% have come from rural area

 -71,44% have come from the urban area

**2. EDUCATIONAL LEVEL** -40% primary education

 -55% medium education

 -5% higher education

**3. HEALTH STATE** -71,4% very good health state

 -12,23% good health state

 -12,23% poor health state

 -0% very poor health state

**4. MEDICAL TREATMENTS** -18,04% have had a regular medical treatment

 (Enalapril,Prednisone,Griseofulvina,Ranitidine)

 - 81,86% have never had a regular medical treatment

**5. ANTECEDENTS OF MUMPS** - 55% no antecedents of mumps

 - 40% with antecedents of mumps

 - 5% unilateral testicular hypertrophy, without antecedents of mumps

**6. NUTRITION STATE** -73,7% normal weight

 -26,3% overweight

**7. TESTICULAR PATHOLOGY** -28,04% of the males have been affected by a testicular pathology of the following types:

-4,76%-varicocele

-4,76%-cystitis

-9,52%-inguinal hernia

-4,76%-gonococcal and syphilitic epididymitis

-81,96% of the males have not suffered from any testicular pathology

**8. THE SMORKERS PROPORTION** -50% smokers

 -50% non-smokers

**9. TYPE OF WATER CONSUMED** -26,64% tap water

 -73,60% sparkling water

**10. THE PROVENIENCE OF THE ALIMENTS** -23,52% ,,BIO,, alimentation

 -76,47% non ,,BIO,, alimentation

**11. NOXIOUS OF THE WORKING PLACE** -85,72% do not work in a toxic environment

 -14,28 work in a toxic environment

 - 4,765 work in a hydrocarbons environment

 - 4,76 work in an environment with cement dust

 - 4,76 work in an environment with ionizing radiations

**12. THE MAIN ETIOLOGIES OF SECRETORY AZOOSPERMIA**

 -ectopy-12%

 -mumps orchitis-3%

 -infectious mumps-17%

 -iterative surgeries-4%

 -varicocele-5%

 -unknown-43%

**13. DISTRIBUTION OF SPERM MOBILITY**

 - in infertile males in the study, there is a significant percent (of 40%), the mobile forms percent is in normal limits

**14. THERAPEUTICAL STRATEGY APPLIED TO THE ICSI STUDY GROUP**

 -ICSI spermatic anomalies first intention-51,2%

 -spermatic anomalies with failure or pauci-fecundation by IVF-27,5%

 -failure or pauci-fecunadtion by IVF- 9,7%

 -flagellation dyskinesias (antispermatic antibodies) -1,2%

-epididimar punctures - 5,6%

-differential punctures - 0,3%

-testicular biopsy -1,8%

-sperm auto conservation - 0,7%

## Conclusions

In the last couple of years, the male cause of infertility has been recognized, contrary to the ancestral conception, which stated the fact that the female factor was incriminating exclusive in the couple infertility. In the balance of the evaluation of the couple infertility, the diagnostic file has the role of reuniting the most important anamnestic, clinical and laboratory information, which are able to lead to a male infertility diagnosis, and, where it is possible, to remove the possible cause through a treatment which is targeted so that IVF is not the first option, due to the fact that it is an invasive and expensive method.

The study has also underlined some risk factors that should be avoided in the period the couple wishes to have babies, among which the following should be mentioned: inadequate alimentation, which leads to obesity, smoking, drugs abuse, or working in a toxic environment.

The start that ICSI has taken at the beginning of 1990 has revolutionized the treatment of male infertility and has allowed many males who were considered infertile, to have a child, by having only a few normal spermatozoos in their sperm.

The initial enthusiasm to recourse to IVF has reduced, despite some positive results, due to the fact that the failure of fecundation is unpredictable and without any apparent reasons, because one could not make the difference between a fertilizing sperm and another which did not have this property; ICSI being able to solve this problem to some extent.
